# Sustainable employability and work engagement: a three-wave study

**DOI:** 10.3389/fpsyg.2023.1188728

**Published:** 2023-06-16

**Authors:** Sait Gürbüz, Arnold B. Bakker, Evangelia Demerouti, Evelien P. M. Brouwers

**Affiliations:** ^1^Tranzo, Scientific Center for Care and Wellbeing, Tilburg School of Social and Behavioral Sciences, Tilburg University, Tilburg, Netherlands; ^2^International Business School, Hanze University of Applied Sciences, Groningen, Netherlands; ^3^Center of Excellence for Positive Organizational Psychology, Erasmus University Rotterdam, Rotterdam, Netherlands; ^4^Department of Industrial Psychology and People Management, University of Johannesburg, Johannesburg, South Africa; ^5^Department of Industrial Engineering and Innovation Sciences, University of Eindhoven, Eindhoven, Netherlands

**Keywords:** sustainable employability, capability approach, work engagement, task performance, job satisfaction

## Abstract

**Introduction:**

The recent concept of sustainable employability (SE), which refers to being able and enabled to achieve valuable work goals, has lately attracted substantial attention in many developed countries. Although limited cross-sectional studies found that SE in the form of capability set was positively associated with work outcomes, why and through which mechanism SE is related to crucial work outcomes remains still unexplored. Therefore, the present three-wave study aimed to (1) investigate the SE-work outcomes linkage over time, and (2) uncover the psychological pathway between SE and two work outcomes (i.e., task performance and job satisfaction) by proposing work engagement as a mediator.

**Methods:**

To test the mediation process, we approached CentERdata to collect data among a representative sample of 287 Dutch workers. We used a three-wave design with approximately a 2-month time lag.

**Results:**

The results of bootstrap-based path modeling indicated that SE was a significant predictor of task performance but not job satisfaction over time. Work engagement mediated the relationships between SE and (a) task performance and (b) job satisfaction.

**Discussion:**

These findings suggest that organizations may foster workers’ task performance and job satisfaction by configuring a work context that fosters SE–allowing workers to be able and be enabled to achieve important work goals.

## Introduction

Owing to the aging population (OECD, 2021) and the shortage of competent young employees, the concept of sustainable employability (SE), has lately attracted substantial attention–particularly in many developed countries (Gürbüz et al., 2022a). Since an aging workforce is more prone to experience age-related health complaints, enhancing a worker’s SE is an important topic from both organizational and workers’ perspectives (Truxillo et al., 2015). From an organizational perspective, a sustainable workforce is functional in reducing the costs of burnout, sickness absenteeism, and personnel turnover (de Jonge and Peeters, 2019). From a worker’s perspective, the topic is also vital because lack of employability may lead to job loss, which in turn, impairs workers’ wellbeing and general health (Berntson and Marklund, 2007).

Although several definitions of SE exist in the literature (see for review; Van der Heijden et al., 2016; Le Blanc et al., 2017; Fleuren et al., 2020; Deng et al., 2021), one of the most recognized conceptualizations of SE has been introduced by Sen’s (1993) based on Van der Klink et al. (2016) capability perspective:

Sustainable employability means that, throughout their working lives, workers can realize tangible opportunities in the form of a set of capabilities. They also enjoy the necessary conditions that allow them to make a valuable contribution through their work, now and in the future, while safeguarding their health and welfare. This requires, on the one hand, a work context that facilitates them, and on the other hand, the attitude and motivation to exploit these opportunities (p. 74).

This conceptualization differs considerably from other definitions in that it integrates the values and capabilities of an employee and the opportunities supplied by the organization (Gürbüz et al., 2022b). The new SE model posits that an individual worker experiences a high degree of SE if he or she (a) considers certain work goals (e.g., involvement in important decisions) as valuable (importance element), (b) has suitable working conditions to accomplish these valuable goals (enablement element), and (c) can realize these goals (ability element, Van der Klink et al., 2016). These three elements are used to assess to what extent the work values are achieved, also called the capability set, which is a proxy tool to measure a worker’s SE (Gürbüz et al., 2022b).

One of the assumptions of the new SE model is that possessing a larger capability set (having work opportunities to fulfill the valued goals and being able to achieve them) or a higher level of SE leads to desirable work outcomes such as wellbeing, work performance, and work (Van der Klink et al., 2016). Indeed, some studies have reported preliminary evidence for this proposition. For instance, Abma et al.’s (2016) found that a larger SE in the form of capability set was positively associated with work performance and work ability, and negatively related to sickness absence. Using a sample of workers with multiple sclerosis as well as a general population, Van Gorp et al. (2018) reported that the associations between SE and (a) work outcomes (e.g., work ability) and (b) health consequences (e.g., fatigue) were stronger for workers who suffered from multiple sclerosis. More recently, Gürbüz et al. (2022a) found that SE was a positive predictor of important work outcomes such as work ability, job satisfaction, and task performance. However, these three studies on the relationships between SE and work outcomes adopted a cross-sectional design, which prevents causal inference. In addition, why and through which mechanism SE impacts crucial work outcomes remains unexplored in the literature.

The present three-wave study has three major aims. First, we aim to investigate whether SE is associated with work outcomes over time. We focus on job satisfaction and task performance because these two work outcomes have substantial implications for organizational performance and workers’ wellbeing (de Beer, 2021). Second, we aim to uncover the psychological pathway between SE and work outcomes. More specifically, integrating the SE model (Van der Klink et al., 2016) with job demands-resources (JD-R) theory (Bakker et al., 2023), we investigate whether work engagement, which refers to high levels of vigor, dedication, and absorption a worker experiences at work (Schaufeli and Bakker, 2023), mediate the relationships between SE and (a) task performance and (b) job satisfaction. We suggest work engagement as a mediator because, according to JD-R theory, work engagement is a crucial motivational pathway between job resources (e.g., supportive supervision, autonomy, skill variety) and desirable work outcomes (e.g., task performance), which is also known as the motivational process to performance (Bakker et al., 2023).

**FIGURE 1:**
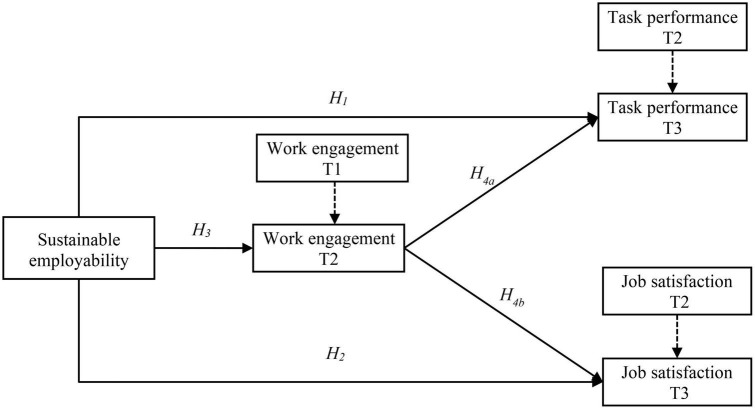
Proposed model of sustainable employability and work outcomes. Dashed lines show the how we controlled for previous levels of the mediator and dependent variables. T, Time.

We intend to make three contributions to the literature. First, cross-sectional studies have indicated that SE in the form of capability set is related to enhanced job satisfaction (Gürbüz et al., 2022a) and task performance (Abma et al.’s, 2016). Building on these studies, we have collected data using three waves to investigate the impact of SE on task performance and job satisfaction, which allows us to see whether the associations explored in earlier studies also hold over time. Second, unifying JD-R theory (Bakker et al., 2023) and the SE model (Van der Klink et al., 2016). We contribute to the literature on SE by proposing that work engagement may explain why a higher SE leads workers to perform well and be satisfied with their jobs. Such knowledge advances our understanding of the psychological pathway between SE and work outcomes. Finally, we add to the nomological network of JD-R theory (Bakker et al., 2023) by investigating SE as a potential antecedent of work engagement, meaning that SE in the form of capability set may be considered as a key resource that shapes workers’ motivation for optimal functioning at work. More explicitly, we argue that being able to carry out the esteemed aspects of work (i.e., ability element of SE), like other personal resources (e.g., self-efficacy), may lead to greater motivation and thus more energy, dedication, and absorption during work (Deci et al., 2017). Furthermore, having suitable work opportunities to realize work goals (enablement element of SE), like other job resources (e.g., organizational support), may encourage employees to be more engaged in their roles. The results of the present research may further help organizations that want to foster workers’ job satisfaction and task performance through influencing SE and work engagement. Figure 1 shows the hypothesized conceptual model of SE and work outcomes.

## Theoretical framework and hypotheses

### Sustainable employability and work outcomes

Building on the capability approach (CA), the new SE model proposes that optimal functioning and wellbeing at work can be achieved when workers possess a set of capabilities (Van der Klink et al., 2016). The concept of capability is defined as the freedom or opportunities that employees have to fulfill “beings or doings people have reason to value” (Sen, 1993, 10). This concept can be explained using the analogy of cycling (Alkire, 2005) to illustrate its elements and functions more clearly. In order to cycle optimally, an individual needs four essential components: a bicycle, the necessary physical abilities, an appropriate physical setting (e.g., a road), and a suitable social setting (e.g., lack of a curfew). The individual cannot cycle if any of these components are absent. Similarly, the CA theorizes that when employees possess a captivating job (bicycle), personal resources (the necessary physical abilities), and can operate in a conducive work environment (the appropriate physical and social setting), they can realize their important work goals to attain optimal functionality at work. Specifically, “capability” alludes to the potential to use cycling as a mode of transportation, whereas “functioning” refers to the choice of cycling rather than other modes of transportation (Gürbüz et al., 2022b). It is crucial that the individual values cycling positively for it to be a feasible capability. The key assertion of the CA is that attention should be paid to what employees value in terms of work goals, the opportunities that allow them to achieve the valued choices, and what they are capable of Van der Klink et al. (2016).

According to the SE model based on CA, workers are more likely to have a larger capability set or a higher level of SE when they are able (i.e., having capacity) and enabled (i.e., having adequate work opportunities) to fulfill certain work goals that they find meaningful. In their exploratory study, Abma et al.’s (2016) investigated a set of seven work goals for the model: “the use of knowledge and skills, development of knowledge and skills, involvement in important decisions, building and maintaining meaningful contacts at work, setting your own goals, having a good income, and contributing to something valuable” (p. 10). These work values are at the heart of the new SE model and are considered meaningful work goals by workers who believe such goals are worthwhile of pursuing (Gürbüz et al., 2022b). Taken together, the model delineates that SE calls for workers to possess the necessary abilities to achieve their work values and, at the same time, a suitable work context that enables them to do so.

Task performance, also called in-role performance, refers to required task-related behaviors that a worker performs to contribute to the goal of an organization (Borman and Motowidlo, 1997). The Ability-Motivation-Opportunity (AMO) framework posits that workers demonstrate high performance when they have the ability (e.g., required skills) (A), are motivated (e.g., rewarding for desired behaviors) (M), and have adequate opportunities (e.g., empowerment) (O) at work (Appelbaum et al., 2000). In other words, the combination of these three distinct elements determines the overall performance of individuals (Boxall and Macky, 2009). The ability component of the AMO model can include both cognitive and physical skills that are necessary to complete a task. The motivation component refers to the psychological drive that inspires an individual to use their capabilities to achieve their task-related goals. Finally, the opportunity component refers to the availability of resources and conditions that facilitate performance. These opportunities may include access to training, adequate job resources, and supportive work environments (Kehoe and Wright, 2013). Taken together, the AMO framework posits that individuals who are more talented, highly motivated, and have access to opportunities are more likely to be successful in the workplace.

Having a higher level of SE in the form of capability set means that workers possess the required ability and suitable work opportunities to achieve the valued aspects of work (Van der Klink et al., 2016). Hence, based on the central tenet of the AMO framework, we argue that when workers have greater SE, they are more likely to demonstrate optimal task performance. “Involvement in important decisions,” for example, is one of the seven work goals in the new model of SE. If an individual worker believes that they are personally able (A) and hold a job that provides work opportunities (O) to realize this goal (M), they are likely to perform well. In line with this assertion, earlier cross-sectional studies (Abma et al.’s, 2016; Gürbüz et al., 2022a) have found that a larger SE is positively related to enhanced task performance. Here, we suggest this association over time because having the capabilities and opportunities to contribute to something valuable will put individuals in a positive spiral of resource accumulation where previous resources (capabilities and opportunities) help individuals to gain more resources over time (Hobfoll et al., 2018). We propose that a higher SE will be positively related to task performance over time.

*Hypothesis 1: SE is positively associated with task performance over time.*

Job satisfaction is defined as “a pleasurable or positive emotional state resulting from the appraisal of one’s job or job experiences” (Locke, 1976, p. 1304). Compared to work engagement, job satisfaction is also a positive indicator of work-related wellbeing but it is lower in activation. Job satisfaction remains a crucial construct for occupational health research because satisfaction with work induces desirable outcomes (e.g., organizational citizenship behavior, Judge et al., 2017), while dissatisfaction leads to negative consequences (e.g., turnover; Griffeth et al., 2000).

Job demands-resources theory provides an important framework for understanding how job demands and job resources can interact to facilitate employee engagement and performance (Bakker et al., 2023). JD-R theory proposes that all job facets can be categorized into two unique groups: job demands and job resources. Job demands (e.g., work pressure) refer to job characteristics that necessitate sustained effort and therefore deplete psychological resources such as vigor and cognitive capacity. In contrast, job resources (e.g., supportive work context, and skill variety) are defined as job facets that have motivational potential, are effective in fulfilling work goals, and can be used to deal with job demands. The theory posits that employee wellbeing and performance are the results of a dynamic interplay between job demands and job resources. For example, job resources satisfy basic psychological needs and therefore increase various aspects of wellbeing, including work engagement and job satisfaction (Bakker et al., 2023). Additionally, according to the theory, when job demands are high, job resources can help to mitigate the strain associated with these demands to sustain high levels of work engagement. Similarly, when job resources are low, job demands can lead to increased strain and decreased work engagement (Bakker et al., 2023). In line with the core prepositions of JD-R theory, prior research has pointed out that a rise in job resources led to an increase in work engagement (Van Wingerden et al., 2018) and job satisfaction (Tims et al., 2013; de Beer et al., 2016). Therefore, it is plausible to anticipate that when workers feel that they can use their abilities (ability element of the SE) and have freedom and opportunities at work (enablement element of the SE) to fulfill their work values, they tend to be enthusiastic about their jobs and are likely to experience job satisfaction. One previous study (Gürbüz et al., 2022b) found cross-sectional evidence for such a relationship. Based on the SE model, we propose:

*Hypothesis 2: SE is positively associated with job satisfaction over time.*

### Sustainable employability and work engagement

Work engagement refers to “a positive, fulfilling, work-related state of mind that is characterized by vigor, dedication, and absorption” (Schaufeli et al., 2002, p. 74). One of the central assumptions of JD-R theory (Bakker et al., 2023) is that personal resources (e.g., self-efficacy, optimism) and job resources (e.g., supportive supervision, autonomy) foster work engagement and eventually result in desirable work outcomes (e.g., task performance) by triggering workers’ intrinsic and extrinsic drive. For example, when workers experience development opportunities at work, they tend to be internally motivated because such job resources satisfy the basic need for competence by enhancing their growth and learning (Deci et al., 2017). Similarly, such work characteristics may also stimulate their extrinsic motivation by making it easier to achieve work-related goals (Bakker et al., 2023).

Integrating JD-R theory (Bakker et al., 2023) with the new SE model (Van der Klink et al., 2016), we contend that when employees are capable of (ability element) and have adequate work opportunities (enablement element) to fulfill their work values, they are more prone to feel motivated, which results in increased vigor, dedication, and immersion at work. This is because being capable means that employees have the personal resources needed to be energized, passionate, and absorbed in their work. Being capable and feeling resourceful stimulates workers’ intrinsic (e.g., fulfilling the essential need for competence) and extrinsic (e.g., facilitating the achievement of work goals) motivation (Deci et al., 2017). Likewise, possessing relevant opportunities to achieve the valued aspects of work means that employees are enabled, just like the availability of job resources, enablement may foster employees’ energy and enthusiasm for their job (i.e., their work engagement). Therefore, we propose:

*Hypothesis 3: SE is positively associated with work engagement over time.*

### Mediating role of work engagement

The linkage between work engagement and task performance is already well-documented in the literature. Since engaged workers experience more positive emotions (e.g., enthusiasm, immersion, optimism) and are often more receptive to exploring novel opportunities (Cropanzano and Wright, 2001; Fredrickson, 2013), they are more likely to find creative solutions for work problems and perform better than those who are not engaged. Extant studies indicated that work engagement is a crucial predictor of self-, supervisor-, and peer-rating of task performance (Bakker et al., 2012; Neuber et al., 2022). It is also reasonable to expect that workers who are engrossed in their tasks and display enthusiasm really enjoy their work and experience a high level of job satisfaction (Fisher, 2000). Engaged workers find their job fascinating, meaningful, and motivating, and they experience pleasant feelings, including pleasure and excitement (Bakker and Demerouti, 2008). Such emotions tend to boost their job satisfaction at work (Fisher, 2000). Therefore, it makes sense to anticipate that workers who have a lot of energy, are excited about what they do, and are thoroughly absorbed in their tasks have a higher likelihood of evaluating their job positively and feeling satisfaction with their job.

Given that work engagement acts as a key process mechanism between job resources and work outcomes (Bakker et al., 2022), building on the motivational process in JD-R theory (Bakker et al., 2023), and the SE model (Van der Klink et al., 2016), we propose that the associations between SE and the work outcomes are mediated by work engagement. More specifically, workers who possess a larger SE (i.e., being capable and having work opportunities to achieve the work goals) are more likely to feel highly engaged, which, in turn, helps workers to demonstrate enhanced task performance and be more satisfied with their job. This is because when workers’ needs are satisfied by organizational supplies (needs–supplies fit), this helps workers to experience more positive attitudes and be successful at work. Indeed, in their meta-analysis, Kristof-Brown et al. (2005) reported that the needs–supplies fit had the largest positive effect on employee work attitudes such as job satisfaction. Following the motivational process in JD-R theory, we argue that if workers believe that their organization supplies suitable work opportunities to fulfill their work values (needs–supplies fit), they tend to experience positive feelings toward their work. In turn, they perform well and are more satisfied with their jobs as their needs are met by environmental supplies. Therefore:

*Hypothesis 4: Work engagement mediates the relationships between SE and (a) task performance and (b) job satisfaction over time.*

## Materials and methods

### Study population and procedure

We collected data over a 6-month period utilizing a three-wave design between the measures of September 2021 and February 2022 via the Longitudinal Internet Studies for the Social Sciences (LISS) panel directed by CentERdata. The LISS panel is comprised of a representative sample of Dutch citizens who participate in monthly Internet surveys. Panel participants were chosen from the population registry using a random selection approach (Scherpenzeel and Das, 2010). Each year, panel members take part in a longitudinal survey concerning a variety of topics. Please visit www.lissdata.nl for further details on the LISS panel.

To minimize potential common method bias (CMB), data were gathered in consecutive three waves (Podsakoff et al., 2012). Although there is no fixed rule for optimal time lags in occupational health research (Griep et al., 2021), earlier research on the topic has shown that depending on contexts, the trajectory of work attitudes can be captured in the short term (Demerouti et al., 2012). Thus, in the present study, a short time lag of approximately a 2-month interval between the three waves was used.

During the first wave (T1, in September 2021), an online survey was sent to randomly chosen LISS panelists who worked for various organizations (*N* = 597). In this wave, the panel members were requested to complete a questionnaire regarding the independent variable (i.e., SE questionnaire), mediator (i.e., work engagement T1), and demographic questions. A total of 401 participants completed the online survey, with a response percentage of 67.2. Since 37 respondents only completed demographic questions, we decided to delete those surveys from the sample. After discarding unfinished surveys 364 usable questionnaires were collected.

A follow-up survey was sent to those participants at the second wave (T2, in November 2021), and 315 out of 364 workers responded (response rate: 86.5%). After discarding incomplete surveys, 305 viable surveys were collected. In this phase, they were asked to fill out a questionnaire regarding the mediator variable (i.e., work engagement T2) again and the outcome variables (i.e., task performance T2 and job satisfaction T2).

At the third wave (T3, in January 2022), a final questionnaire was sent to the participants who responded to the T2 survey, and 290 out of 305 employees filled out the questionnaires (response rate = 95.1%). In this wave, participants were requested to complete a questionnaire regarding the outcome variables again (i.e., task performance and job satisfaction). In the final wave, we decided to discard three respondents as they failed to complete items regarding task performance. Considering the number of respondents in the first wave, the final response rate was 48.5%. The final sample included 287 respondents who answered the three surveys after deleting incomplete ones. The questionnaire data were matched via each respondent’s unique panel number.

Non-response tests between T1 and T3 showed that no significant differences were observed on main variables, including SE [*t*(362) = 0.01, *p* = 0.992], work engagement [*t*(362) = 0.57, *p* = 0.572]. Out of 287 respondents, 53.3% were female (*N* = 159), and the mean age was 46.59 years (*SD* = 12.33), with an average organizational tenure of 13.07 years (*SD* = 11.61). The majority of the respondents held an intermediate vocational degree or above (77.3%, *N* = 222) and were employed in a profit organization (56.1%, *N* = 161).

### Measures

#### The capability set for work questionnaire (CSWQ)

Developed by Abma et al.’s (2016) was used to measure SE. This tool is an index that determines the extent to which seven work goals (i.e., “the use of knowledge and skills, development of knowledge and skills, involvement in important decisions, building and maintaining meaningful contacts at work, setting your own goals, having a good income, and contributing to something valuable”) (p. 10), are considered valuable by a worker, are enabled in the workplace, and can be achieved. For each work aspect, respondents were asked “How important is the work value for you?”, “Does your work provide the opportunities to achieve this work value?” and “To what extent are you able to actually achieve this work value?” on a scale from 1 (“not at all”) to 5 (“a very large extent”). Consistent with previous studies (e.g., Abma et al.’s, 2016; Gürbüz et al., 2022b), the mean score of 21 questions was calculated to measure works’ SE. A high SE value indicates a worker has a higher SE score. The CSWQ can be found in Supplementary Appendix 1.

#### Work engagement

Was rated with the three-item ultra-short Utrecht Work Engagement Scale (UWES) (Schaufeli et al., 2006, 2019). A sample item is “I am enthusiastic about my job.” The responses were provided on a five-point scale ranging from 1 (“never”) to 5 (“very often”). Earlier research has shown that this abbreviated version is a parsimonious measure of work engagement that may be used in place of the lengthier version (Schaufeli et al., 2019). Cronbach’s α of the scale was 0.86 at T1 and 0.87 at T2. Test-retest reliability of work engagement between the times was also good (*r* = 0.73).

#### Task performance

Was rated with three items by workers (Pettit et al., 1997). The items are “how would > you, your direct supervisor, and your colleagues > evaluate your current overall work performance?” Items were rated on a five-point scale, ranging from 1 (“very poor”) to 5 (“excellent”). Cronbach’s α of the scale was 0.87 at T2 and 0.88 at T3. Test-retest reliability of the measure between T2 and T3 was also high (*r* = 0.71).

#### Job satisfaction

Was assessed using one overall item (Dolbier et al., 2005): “Taking everything into consideration, I am satisfied with my job.” The response was given on a seven-point scale (1 = “strongly disagree,” 7 = “strongly agree”). Previous research has demonstrated that a single-item measure can assess job satisfaction in a valid way (Wanous et al., 1997). Test-retest reliability of the job satisfaction measure between T2 and T3 was good (*r* = 0.68).

#### Controls

Since previous research has indicated that age and gender influence the outcome variables (Jung et al., 2007), these variables were employed as controls.

### Analytical strategy

SPSS 28 and AMOS 28 for Windows were used to analyze the data. Our data analysis strategy is composed of several steps. First, we evaluated the factor structure of work engagement and task performance by performing a series of confirmatory factor analyses (CFAs) with the Maximum Likelihood estimation method. Further, we used Harman’s one-factor test (Podsakoff et al., 2012) to assess the presence of CMB. Next, we assessed the longitudinal invariance of work engagement and task performance scales to determine if they were completed consistently across the waves (Horn and McArdle, 1992). To test invariance, we compared an unrestricted model (i.e., factor loadings were allowed to be freely calculated across the waves) with a restricted one (i.e., factors loadings were restricted to be equal across the waves). The scales were considered invariant if the *X*^2^ difference between the models was non-significant (Vandenberg and Lance, 2000). Given that the CSWQ is an index-type measure, on the one hand, job satisfaction was measured with the one-overall item, on the other hand, we did not include these two constructs in the measurement validation analysis.

In the last step, we tested the research hypotheses by conducting bootstrap-based structural equation modeling (SEM) based on 5,000 resamples. Since the CSWQ is an index-type measure, the SEM was employed based on manifest variables (i.e., average scores of constructs), with a cross-lagged panel design (Wang et al., 2017), meaning that earlier measurements of the repeated measures were included. This allowed us to control for the previous effect of the mediator and the outcome variables. The indirect effects were evaluated as significant if a 95 percent bias-corrected bootstrap confidence interval (CI) does not include zero (Hayes, 2022). We evaluated CFAs and SEM models based on χ^2^*/df* (degree of freedom), RMSEA (root-mean-squared error of approximation, CFI (comparative fit index); and SRMR (standardized root mean squared residual) (Kline, 2015). χ^2^/*df* < 5, RMSEA < 0.08, CFI > 0.90, SRMR < 0.08 were considered acceptable fit indices (Hu and Bentler, 1999).

## Results

### Measurement validation and the longitudinal invariance of the scales

We initially tested the constructs’ factor structure (with work engagement at T1 and T2 and task performance at T2 and T3) by performing a CFA. As suggested by Pitts et al. (1996), measurement errors of either work engagement or task performance items (i.e., items indicating the same constructs) across the waves were let to covary. The result of the CFA indicated that the measurement model fit the data well, χ2 = 80.66, df = 44, CFI = 0.98, RMSEA = 0.05, SRMR = 0.04. Next, we evaluated if CMB might cause a threat to our study. Since a one-factor model (i.e., all items in the three waves were included) yielded a poor fit with the data, χ2 = 956.56, df = 54, CFI = 0.59, RMSEA = 0.24, SRMR = 0.21, we considered that CMB was not a serious issue in our data (Podsakoff et al., 2012). Finally, we assessed the longitudinal invariance of work engagement and task performance scales by comparing the unconstrained models (i.e., factor loadings were allowed to be freely calculated over the waves) with the constrained ones in which factor loadings were limited to be equal over the waves. The results pointed out that work engagement (Δχ^2^/Δdf = 1.01/2, *p* = 0.60) and task performance (Δχ^2^/Δdf = 4.33/2, *p* = 0.12) were invariant over time because the χ^2^ differences between the models were found non-significant. Collectively, these results revealed that work engagement and task performance constructs were conceptually distinct and measured consistently over time.

### Descriptive statistics

Table 1 depicts descriptive statistics and Pearson’s correlation coefficients among the variables. T1 SE was positively associated with T2 work engagement (*r* = 0.37, *p* < 0.01), T3 task performance (*r* = 0.24, *p* < 0.01), and T3 job satisfaction (*r* = 0.34, *p* < 0.01). In addition, T2 work engagement was positively associated with T3 task performance (*r* = 0.41, *p* < 0.01) and T3 job satisfaction (*r* = 0.66, *p* < 0.01). The results also show that the three separate components, like the mean score of SE, were positively associated with the mediator and the outcomes. Regarding the demographics, the SE score was not correlated with age (*r* = −0.02, *p* = 0.79), while it was weakly negatively associated with gender (*r* = −0.13, *p* < 0.05), indicating that compared to males, females reported lower levels of SE. Finally, Table 1 demonstrates that age was positively correlated with T1 and T2 work engagement as well as T3 job satisfaction, meaning that older employees were more engaged and satisfied with their job than younger employees.

**TABLE 1:** Means, standard deviations, Pearson’s correlations among the variables (*N* = 287).

Variables	Mean	SD	1	2	3	4	5	6	7	8	9	10	11
1. SE (T1)	3.53	0.54	-										
2. Importance dimension (T1)	3.77	0.55	0.80**										
3. Opportunity dimension (T1)	3.48	0.62	0.92**	0.59**									
4. Ability dimension (T1)	3.41	0.61	0.93**	0.61**	0.91**								
5. Work engagement (T1)	3.58	0.69	0.40**	0.21**	0.44**	0.44**	-						
6. Work engagement (T2)	3.67	0.67	0.37**	0.23**	0.41**	0.39**	0.73**	-					
7. Task performance (T2)	3.72	0.60	0.23**	0.27**	0.14*	0.21**	0.39**	0.32**	-				
8. Task performance (T3)	3.73	0.63	0.24**	0.27**	0.18**	0.23**	0.32**	0.41**	0.71**	-			
9. Job satisfaction (T2)	5.53	1.28	0.44**	0.18**	0.55**	0.49**	0.61**	0.53**	0.20**	0.21**	-		
10. Job satisfaction (T3)	5.56	1.22	0.34**	0.16**	0.44**	0.38**	0.57*	0.66**	0.20**	0.24**	0.68**	-	
11. Gender (1 = male, 2 = female)	-	-	-0.13*	-0.05	-0.15*	-0.15**	-02	-0.00	-0.11	-0.09	-0.02	-0.02	-
12. Age	46.59	12.34	-0.02	-0.09	0.01	0.01	0.13*	0.12*	0.08	0.08	0.11	0.15*	-0.04

*p < 0.05; **p < 0.01 (two-tailed). SE, sustainable employability; SD, standard deviation.

### Hypotheses testing

We tested the research hypotheses by running two independent structural path models: M1 (direct effects–unmediated model) and M2 (mediation model). M1 was used to test Hypothesis 1 and Hypothesis 2, while M2 was utilized to test the other hypotheses. We defined the temporal stability routes of the meditator and the outcomes for both M1 and M2, meaning that the previous levels of the mediator and dependent variable were controlled for. Thus, T1 work engagement predicted T2 work engagement; T2 job satisfaction and task performance predicted T3 job satisfaction and T3 task performance, respectively. In addition, gender and age were included as controls in the models.

Table 2 shows the results of the path models. Our first two hypotheses proposed that SE would be positively associated with task performance (Hypothesis 1) and job satisfaction (Hypothesis 2) over time. M1 provided a good fit to the data (χ2*/df* = 1.70; RMSEA = 0.05; CFI = 0.99; SRMR = 0.03). As can be seen in the table, controlling for T2 task performance, T1 SE significantly predicted T3 task performance (β = 0.09, 95% *CI* = 0.02 to 0.18), supporting Hypothesis 1. However, T1 SE did not predict T3 job satisfaction measured (β = 0.05, 95% *CI* = −0.02 to 0.29) while controlling for the previous effect of T2 job satisfaction. This means that Hypothesis 2 was not supported.

**TABLE 2:** The results of the unmediated (M1) and the mediated model (M2).

Paths	Estimate	SE	*P*	95 % CI	
				**Lower**	**Upper**
**Model 1 (The unmediated model)**					
**Cross-lagged effects**					
T1 Sustainable employability = > T3Task performance	0.09	0.05	*	0.02	0.18
T1 Sustainable employability = > T3Job satisfaction	0.05	0.11	0.27	-0.02	0.29
**Temporal stability effects**					
T2 Task performance = > T3Task performance	0.69	0.05	***	0.62	0.75
T2 Job satisfaction = > T3Job satisfaction	0.65	0.05	***	0.54	0.74
**Model 2 (The mediated model)**					
**Cross-lagged effects**					
T1 Sustainable employability = > T2Work engagement	0.10	0.07	*	0.01	0.19
T2 Work engagement = > T3Task performance	0.20	0.05	***	0.10	0.30
T2 Work engagement = > T3Job satisfaction	0.42	0.09	***	0.30	0.54
T1 Sustainable employability = > T3Task performance	0.02	0.07	0.61	-0.07	0.14
T1 Sustainable employability = > T3Job satisfaction	0.02	0.12	0.60	-0.10	0.05
**Temporal stability effects**					
T2 Task performance = > T3Task performance	0.64	0.04	***	0.55	0.72
T2 Job satisfaction = > T3Job satisfaction	0.47	0.04	***	0.35	0.60
T1 Work engagement = > T2Work engagement	0.69	0.04	***	0.62	0.75
**Indirect effects**	**Effect**	**Boot SE**	***P***	**95 % Boot CI**	
				**Lower**	**Upper**
Sustainable employability = > Work engagement = > Task performance	0.02	0.02	*	0.01	0.04
Sustainable employability = > Work engagement = > Job satisfaction	0.04	0.01	*	01	0.08

*p < 0.05; ***p < 0.001 (two-tailed). SE, standard error; Standardized estimates were reported. Age and gender were controlled for both models. A 95 % bias-corrected bootstrap was used for the indirect effects.

**FIGURE 2:**
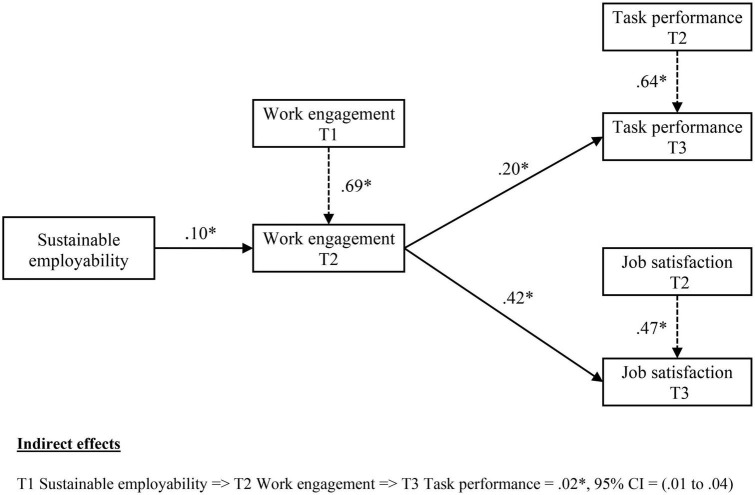
SEM results of the mediated model of sustainable employability and work outcomes. T, Time; CI, confidence interval; * Zero not included in 95% bias-corrected bootstrap CI; Lower and upper CIs were given in parentheses; Standardized betas were reported (gender and age were controlled); Direct effects between SE and the outcomes were insignificant and left out for simplicity.

The remaining research hypotheses were assessed using M2 (i.e., mediation model), which provided a good fit to the data (χ^2^*/df* = 2.21; RMSEA = 0.06; CFI = 0.98; SRMR = 0.05). Figure 2 shows summary results for M2. Hypothesis 3 postulated that SE would be related to increased work engagement over time. As expected, the positive association between T1 SE and T2 work engagement was significant (β = 0.10, 95% *CI* = 0.01 to 0.19) while controlling for the effect of T1 work engagement. Therefore, Hypothesis 3 was supported.

Our final hypothesis postulated that SE would be indirectly associated with (a) task performance and (b) job satisfaction through work engagement. As anticipated, bias-corrected bootstrap estimation results with 5,000 resamples indicated that the indirect effects of T1 SE on T3 task performance (estimate = 0.02, 95% *CI* = 0.01, 04) and job satisfaction (estimate = 0.04, 95% *CI* = 0.01, 08) through T2 work engagement were significant (see Figure 2 and Table 2) while controlling for the previous levels of the mediator and outcomes. This indicates that work engagement mediated the relationships between SE and the two work outcomes, providing support for Hypothesis 4.

Additionally, we have alternatively examined the various two-way and three-way interaction effects of SE components (i.e., importance × ability, importance × opportunity, and importance × ability x opportunity). However, as can be seen in Table 3, we found no significant interaction effects although several main effects of the SE component are significant, suggesting that the impact of each aspect doesn’t depend on the level of the other aspects.

**TABLE 3:** Results of alternative analyses.

Dependent variables									
**Predictors**	**Work engagement** **(T2)**			**Job satisfaction** **(T3)**			**Task performance** **(T3)**		
	***B***	***SE***	***p***	***B***	***SE***	***p***	***B***	***SE***	***p***
**Results of regression analyses showing interaction effects**									
Importance	0.01	0.09	0.96	-0.28	0.16	0.07	0.26	0.08	**
Ability	0.43	0.07	***	0.91	0.14	***	0.08	0.07	0.26
Importance X Ability	0.09	0.08	0.27	-0.02	0.15	0.92	0.11	0.08	0.18
Importance	0.02	0.08	0.80	-0.33	0.15	*	0.31	0.08	***
Opportunity	0.43	0.07	***	1.03	0.13	***	0.02	0.07	0.73
Importance X Opportunity	0.14	0.09	0.09	0.05	0.15	0.71	0.10	0.09	0.22
Importance	-0.03	0.10	0.71	0.20	0.18	0.27	0.23	0.10	*
Ability	0.25	0.15	0.11	-0.05	0.28	0.86	0.27	0.15	0.09
Opportunity	0.34	0.15	*	1.01	0.49	***	-0.13	0.15	0.36
Importance X Ability X Opportunity	-0.10	0.09	0.28	-0.09	0.17	0.61	-0.02	0.10	0.84
**Results of path analyses showing the impacts of the seven work values**									
The use of knowledge and skills	0.14	0.05	*	0.48	0.10	***	0.12	0.05	*
Development of knowledge and skills	0.05	0.05	0.26	0.15	0.09	0.10	-0.02	0.05	0.67
Involvement in important decisions	-0.03	0.06	0.64	0.40	0.09	***	0.15	0.05	**
Meaningful contacts at work	-0.02	0.05	0.70	0.49	0.10	***	0.13	0.05	*
Setting your own goals	-0.08	0.05	0.10	0.14	0.08	0.10	0.05	0.05	0.30
Having a good income	0.12	0.04	*	0.13	0.09	0.15	-0.01	0.05	0.78
Contributing to something valuable	0.28	0.05	***	0.32	0.08	***	0.11	0.04	*

*p < 0.05; **p < 0.01; ***p < 0.001; SE, standard error; Unstandardized estimates were reported. Age and gender were controlled.

Furthermore, to evaluate the robustness of our main results, in line with Abma et al.’s (2016), we included the capability set as the seven work values and performed path analyses using the AMOS. Expectedly, the results of the paths analyses indicated significant relationships between various several work values and T2 work engagement, as well as T3 job satisfaction and task performance (see Table 3). These results are consistent with Abma et al.’s (2016) findings, which found that individual capability items (e.g., use of knowledge and skills) were positively related to work outcomes such as work functioning, work performance, and work ability.

## Discussion

Sustainable employability (SE) is nowadays a crucial issue both for organizations and workers due to the aging and overburdened workforce that experiences an increasing number of health problems. Thus, many organizations take steps to foster their workers’ long-term SE. Although limited cross-sectional studies found that SE in the form of capability set was positively associated with work outcomes, why and through which mechanism SE is related to crucial work outcomes remains still unexplored. The present three-wave study, therefore, aimed to (1) investigate whether SE influences work outcomes (i.e., task performance and job satisfaction) over time and (2) discover the psychological pathway between SE and work outcomes by employing work engagement as a mediator. We developed our model by integrating two emergent theories: the SE model building on capability perspective (Van der Klink et al., 2016) and JD-R theory (Bakker et al., 2023). The findings of this three-wave study demonstrated that SE is positively related to task performance but job satisfaction over time. Moreover, our results revealed that T2 work engagement mediated the relationships between T1 SE and T3 task performance and job satisfaction. In other words, workers who possessed greater SE in the form of a capability set experienced more work engagement, in turn, showed higher task performance and were more satisfied with their jobs. These results provide insights into how constituents (i.e., ability and enablement) of the SE act as job and personal resources to enhance work motivation and consequently work outcomes.

The current study offers three main theoretical implications for the literature. First, we showed that a higher level of SE in the form of capability set was related to enhanced work performance over time. Consistent with the results of earlier studies (Abma et al.’s, 2016; Gürbüz et al., 2022b) our findings provide more robust evidence that SE indeed positively impacts task performance of workers over time–although it should be noted that the direct and indirect effects of SE were relatively weak. This finding is also in line with the basic tenet of the AMO model, which posits that workers demonstrate high performance when they have ability, are motivated, and have opportunities at work (Appelbaum et al., 2000; Boxall and Macky, 2009). Our results reveal that possessing the capabilities and opportunities to contribute to something valuable will put individuals in a positive spiral of resource accumulation where previous resources (capabilities and opportunities) help individuals to gain more resources (i.e., showing higher performance) over time (Hobfoll et al., 2018; Bakker et al., 2023). However, unexpectedly, inconsistent with the findings of Gürbüz et al. (2022b), SE was not directly associated with job satisfaction over time. This result implies that, over time, SE only leads to enhanced job satisfaction via higher level of work engagement. The lack of a direct relationship, however, can be partly explained by the fact that we conducted a very conservative test, in which the previous effect of job satisfaction (T2) was controlled for. Thus, although T1 SE was related to T3 job satisfaction, this relationship disappears when trying to predict a unique variance in job satisfaction that does not overlap with T2 job satisfaction. Our findings contribute to the discussion of the consequences of SE as it empirically investigates this linkage over time.

Second, we demonstrated the underlying mechanism between SE and work outcomes. Whereas the consequences of SE have been researched, why possessing larger SE enhances desirable work outcomes has not been explored. Therefore, our study contributes to the new SE model (Van der Klink et al., 2016) as it uncovers a psychological pathway that helps to understand the associations between SE and crucial work outcomes by suggesting work engagement as a mediator. Specifically, integrating JD-R theory (Bakker et al., 2023) with the new SE model, the results of the present study provide empirical evidence that workers who are capable and possess work opportunities to fulfill important work goals (a higher level of SE) are more likely to feel more engaged in their work, in turn, perform better and are more satisfied with their job. These novel findings are also in line with PO fit theory (Edwards et al., 2006) in that if workers believe that their organization supplies suitable work opportunities to fulfill their work goals (needs–supplies fit), they tend to experience positive feelings toward their work. In turn, they perform well and are more satisfied with their jobs as their needs are met by environmental supplies. Therefore, this study expands earlier studies (Hakanen et al., 2008; Van Wingerden et al., 2018) investigating the motivational process (i.e., job resources → work engagement → positive work outcomes) by showing that SE is related to enhanced task performance and job satisfaction through work engagement.

Finally, we add to the nomological network of JD-R theory (Bakker et al., 2023) by suggesting SE as a potential antecedent of work engagement, meaning that SE in the form of capability set may be considered as a new personal and job resource that foster worker’s work engagement for an optimal functioning at work. More precisely, being capable (ability element of SE) to fulfill the valued aspects of work, similar to other personal resources (e.g., self-efficacy) leads workers to be more energized, passionate, and absorbed in their work by stimulating their motivation (Deci et al., 2017). Similarly, having suitable opportunities to the valuable work goals (enablement element of SE), just like the availability of job resources (e.g., organizational support), fosters employees’ energy and enthusiasm in their job (see also, Van Wingerden et al., 2018). As both ability and enablement elements of SE are functional in realizing the valuable work goals, they can function as motivating job characteristics that stimulate work engagement, which is in line with the basic assertion of JD-R theory (Bakker et al., 2023).

Given that our study demonstrates that SE in the form of capability set plays a crucial role in predicting work outcomes via work engagement it has several practical implications as well. Organizations that aim to foster work engagement, task performance, and job satisfaction of workers should cultivate and invest in workers’ SE level. First, we suggest that organizations use the CSWQ tool (Abma et al.’s, 2016) to diagnose which of the seven work goals (i.e., valued aspects of the work) are considered important by workers. Because the work goals in the CSWQ tool may be unique to each individual worker. For example, “involvement in important decisions” can be an important goal for some employees, while others may not consider this goal valuable.

Second, organizations should determine how well workers are capable of achieving these valuable work goals and whether they have adequate work opportunities to fulfill them (Gürbüz et al., 2022b). Lastly, organizations and managers should focus on work (e.g., autonomy), personal (e.g., self-efficacy), and organizational (e.g., opportunity-enhanced HR practices) factors to foster workers’ SE level by designing work context in a sustainable manner.

Although the present study is the first endeavor to provide evidence on the topic using a representative Dutch sample with a three-wave design it has some limitations. First, all study variables are assessed via self-source rating by workers. Upcoming research can at least use other sources (e.g., supervisor) to measure task performance as other study constructs (i.e., SE, work engagement, and job satisfaction) are best assessed via self-report measures (Sousa-Poza and Sousa-Poza, 2000). Second, we used a three-way time-lagged design to test the psychological pathway between SE and work outcomes, which may have helped in lessening CMB. However, we still did not have control over causality, because this is a field study, not an experiment in which individuals are taught to become more employable (e.g., job crafting interventions). Third, we were not able to include the SE measure in the measurement validation analysis due to the current characteristics (i.e., it a “list” type measure) of the CSWQ, which has thus some repercussions for the factorial validity - although previous research (Gürbüz et al., 2022a) confirmed its convergent and predictive validity. The last limitation is that in the present study, we have focused on the mediation mechanism between SE and the two work outcomes through work engagement. Future studies can advance our understanding of SE-work outcomes linkage if they explore which boundary conditions modify these associations. For instance, do proactive personality and work autonomy amplify the positive associations between SE and work engagement as combined effects of multiple resources are functional in unleashing the motivational element of a job?

## Conclusion

This three-wave study reveals that SE in the form of capability set is an important antecedent of work outcomes through work engagement. When workers are capable and hold a job that enables them to fulfill valuable work goals (i.e., work capabilities) they are more likely to feel more engaged at work. This motivational state helps workers perform better and be more satisfied with their job. Integrating the new model of SE based on capability lens with JD-R theory, our finding suggests that to foster desired work outcomes, organizations should configure job and organizational settings in a sustainable fashion that allows employees to be able and enabled to achieve the valued aspects of their work.

## Data availability statement

The raw data supporting the conclusions of this article will be made available by the authors, without undue reservation.

## Ethics statement

The Ethics Review Board of Tilburg University approved the study design, protocol, and data management plan (Registration Number: RP606). The patients/participants provided their written informed consent to participate in this study.

## Author contributions

SG and EB designed the study. SG wrote the manuscript. AB, ED, and EB contributed to reviewing and revising the manuscript. All authors read and approved the final manuscript.
