# Supplementary material for: Sustainable employability and work engagement: a three-wave study
Source: Front Psychol. 2023 Jun 16;14:1188728. doi: 10.3389/fpsyg.2023.1188728 (PMC10313196; doi:10.3389/fpsyg.2023.1188728)
Supplement: Supplementary file 1 [file Data_Sheet_1.docx]

Supplementary Material

**Sustainable employability and work engagement: A three-wave study**

**Sait Gürbüz *, Arnold B. Bakker, Evangelia Demerouti, Evelien P.M. Brouwers**

**Correspondence:** Corresponding Author: s.g.gurbuz@tilburguniversity.edu

# Supplementary Material

*The Capability Set for Work Questionnaire (CSWQ)*

The following questions address different aspects of your working life. Question a) is about how important the different aspects are for you. Question b) asks about the opportunities in your current employment to realize these aspects. Question c) asks to what extent you actually achieve these aspects in your current employment.

| 1 | ***Using*** *knowledge and skills* | Very little extent  1 | Little extent  2 | Neutral  3 | Large extent  4 | Very large extent  5 |
| --- | --- | --- | --- | --- | --- | --- |
| a | It is *important* for me to be able to use my knowledge and skills at work. | ( ) | ( ) | ( ) | ( ) | ( ) |
| b | My current employment offers me enough *opportunities* to do this. | ( ) | ( ) | ( ) | ( ) | ( ) |
| c | I am *able to* actually achieve this. | ( ) | ( ) | ( ) | ( ) | ( ) |

| 2 | ***Developing*** *knowledge and skills* | Very little extent  1 | Little extent  2 | Neutral  3 | Large extent  4 | Very large extent  5 |
| --- | --- | --- | --- | --- | --- | --- |
| a | It is *important* for me to be able to develop my knowledge and skills at work. | ( ) | ( ) | ( ) | ( ) | ( ) |
| b | My current employment offers me enough *opportunities* to do this. | ( ) | ( ) | ( ) | ( ) | ( ) |
| c | I am *able to* actually achieve this. | ( ) | ( ) | ( ) | ( ) | ( ) |

| 3 | *Being involved in important decisions* | Very little extent  1 | Little extent  2 | Neutral  3 | Large extent  4 | Very large extent  5 |
| --- | --- | --- | --- | --- | --- | --- |
| a | It is *important* for me to be involved in important decisions concerning my work. | ( ) | ( ) | ( ) | ( ) | ( ) |
| b | My current employment offers me enough *opportunities* to do this. | ( ) | ( ) | ( ) | ( ) | ( ) |
| c | I am *able to* actually achieve this. | ( ) | ( ) | ( ) | ( ) | ( ) |

| 4 | *Having or building meaningful working relationships with others* | Very little extent  1 | Little extent  2 | Neutral  3 | Large extent  4 | Very large extent  5 |
| --- | --- | --- | --- | --- | --- | --- |
| a | It is *important* for me to have or build meaningful relationships at work (e.g. with colleagues or clients). | ( ) | ( ) | ( ) | ( ) | ( ) |
| b | My current employment offers me enough *opportunities* to do this. | ( ) | ( ) | ( ) | ( ) | ( ) |
| c | I am *able to* actually achieve this. | ( ) | ( ) | ( ) | ( ) | ( ) |

| 5 | *Setting your own goals* | Very little extent  1 | Little extent  2 | Neutral  3 | Large extent  4 | Very large extent  5 |
| --- | --- | --- | --- | --- | --- | --- |
| a | It is *important* for me to be able to set my own goals at work. | ( ) | ( ) | ( ) | ( ) | ( ) |
| b | My current employment offers me enough *opportunities* to do this. | ( ) | ( ) | ( ) | ( ) | ( ) |
| c | I am *able to* actually achieve this. | ( ) | ( ) | ( ) | ( ) | ( ) |

| 6 | *Earning a good income* | Very little extent  1 | Little extent  2 | Neutral  3 | Large extent  4 | Very large extent  5 |
| --- | --- | --- | --- | --- | --- | --- |
| a | It is *important* for me to be able to earn a good income. | ( ) | ( ) | ( ) | ( ) | ( ) |
| b | My current employment offers me enough *opportunities* to do this. | ( ) | ( ) | ( ) | ( ) | ( ) |
| c | I am *able to* to actually achieve this. | ( ) | ( ) | ( ) | ( ) | ( ) |

| 7 | *Making a meaningful contribution through my work* | Very little extent  1 | Little extent  2 | Neutral  3 | Large extent  4 | Very large extent  5 |
| --- | --- | --- | --- | --- | --- | --- |
| a | It is *important* for me to be able to make a meaningful contribution through my work. | ( ) | ( ) | ( ) | ( ) | ( ) |
| b | My current employment offers me enough *opportunities* to do this. | ( ) | ( ) | ( ) | ( ) | ( ) |
| c | I am *able to* actually achieve this. | ( ) | ( ) | ( ) | ( ) | ( ) |

**Source:** Abma et al. The capability set for work: development and validation of a new questionnaire. *Scand J Work Environ Health* **2016**, *42,* 34-42. doi:10.5271/sjweh.3532.
